# Automated real-time monitoring of human pluripotent stem cell aggregation in stirred tank reactors

**DOI:** 10.1038/s41598-019-48814-w

**Published:** 2019-08-23

**Authors:** Ivo Schwedhelm, Daniela Zdzieblo, Antje Appelt-Menzel, Constantin Berger, Tobias Schmitz, Bernhard Schuldt, Andre Franke, Franz-Josef Müller, Ole Pless, Thomas Schwarz, Philipp Wiedemann, Heike Walles, Jan Hansmann

**Affiliations:** 1 University Hospital Würzburg, Department Tissue Engineering and Regenerative Medicine (TERM), 97070 Würzburg, Germany; 2 Translational Center for Regenerative Therapies, Fraunhofer Institute for Silicate Research ISC, 97070 Würzburg, Germany; 3 University Hospital Schleswig-Holstein, Department of Psychiatry and Psychotherapy, 24105 Kiel, Germany; 4 University Hospital Schleswig-Holstein, Institute of Clinical Molecular Biology, 24105 Kiel, Germany; 5 Fraunhofer Institute for Molecular Biology and Applied Ecology IME, 22525 Hamburg, Germany; 6 Mannheim University of Applied Sciences, Institute of Molecular and Cell Biology, 68163 Mannheim, Germany

**Keywords:** Stem-cell biotechnology, Biomedical engineering

## Abstract

The culture of human induced pluripotent stem cells (hiPSCs) at large scale becomes feasible with the aid of scalable suspension setups in continuously stirred tank reactors (CSTRs). Innovative monitoring options and emerging automated process control strategies allow for the necessary highly defined culture conditions. Next to standard process characteristics such as oxygen consumption, pH, and metabolite turnover, a reproducible and steady formation of hiPSC aggregates is vital for process scalability. In this regard, we developed a hiPSC-specific suspension culture unit consisting of a fully monitored CSTR system integrated into a custom-designed and fully automated incubator. As a step towards cost-effective hiPSC suspension culture and to pave the way for flexibility at a large scale, we constructed and utilized tailored miniature CSTRs that are largely made from three-dimensional (3D) printed polylactic acid (PLA) filament, which is a low-cost material used in fused deposition modelling. Further, the monitoring tool for hiPSC suspension cultures utilizes *in situ* microscopic imaging to visualize hiPSC aggregation in real-time to a statistically significant degree while omitting the need for time-intensive sampling. Suitability of our culture unit, especially concerning the developed hiPSC-specific CSTR system, was proven by demonstrating pluripotency of CSTR-cultured hiPSCs at RNA (including PluriTest) and protein level.

## Introduction

Due to their attributes of unlimited self-renewal ability and multi-lineage-differentiation capacity, human induced pluripotent stem cells (hiPSCs) bear a great potential to generate large numbers of lineage-specific progenies *in vitro*^1,2^. These capabilities and qualities strengthen the role of hiPSCs as an attractive cell source for tissue engineering applications^3–6^. Possible uses include the generation of artificial tissue arrangements for disease modelling and pharmaceutical drug development during efficacy and toxicity assessment^5,7–10^. On a larger scope, previous studies also successfully evaluated the potential of hiPSCs and their progenies for tissue regeneration in cell deficiency scenarios such as spinal cord injury, macular degeneration, type I diabetes, and Parkinson disease^11–14^.

The steady production of sufficient numbers of pluripotent stem cells with clinically relevant quality, however, is still challenging, as the format of conventional adherent monolayer culture is limited to lab-scale production. The use of standard labware such as T- or shake flasks is adequate for small-scale studies and routine use. For clinical applications, however, significantly greater amounts in the order of 10^9^–10^12^ undifferentiated stem cells are required to generate sufficient amounts of differentiated progenies for regenerative purposes^15,16^. For instance, 1–2 × 10^9^ cardiomyocytes are needed to replace damaged cardiac tissue after myocardial infarction^17,18^. Likewise, approximately 1.3 × 10^9^ insulin-producing beta cells are necessary for insulin independence of a 70-kg patient after islet transplantation^19^.

In an attempt to meet such demands, hiPSCs are cultured as three dimensional (3D) scalable cell suspensions^20^. Here, a common approach is to start off in small-scale vessels comprising petri dishes, multi-well plates, or spinner flasks and then transit to continuously stirred tank reactors (CSTRs) of increasing sizes. CSTRs have been the gold standard of the pharmaceutical industry for many decades and are state-of-the-art tools for scalable bioprocesses^21^. Besides their scalability, further advantages include the possibility of full process control and automated instrumentation of physical process parameters such as pH and dissolved oxygen, which ultimately contributes to reproducible and robust bioprocesses.

The format of 3D hiPSC suspension cultures further offers striking advantages over conventional two-dimensional monolayer culture. For instance, the need for extracellular matrix components that are characterized by high batch-to-batch variations is omitted, thereby paving the way for defined culture conditions that are compliant with good manufacturing practice (GMP)^22–24^. However, in the absence of a suitable matrix or scaffold, hiPSC proliferation in suspension is characterized by self-aggregation, and, as a result, the formation of macroscopic spheroids that increase in volume over time^25^. This process is further promoted by the merging of two or more free-floating aggregates. Consequently, nutrient and oxygen concentration gradients are likely to occur that negatively affect cells in the spheroid centre. Likewise, the removal of metabolic waste products is unbalanced, which may ultimately lead to cell starvation, spontaneous differentiation, and necrotic aggregate cores^26,27^. On that score, hiPSC aggregation under suspension culture conditions is a very important process parameter that demands specific monitoring.

Taken together, we identified five key requirements that a hiPSCs-specific CSTR ought to meet: First, hiPSC CSTRs should allow the generation of high-quality stem cells exhibiting a pluripotent nature in the most robust fashion **(req. I)**. Second, the reactor system needs to be versatile in scaling to efficiently adapt the production process to the output demands and consequently the bioreactor cascading **(req. II)**. Third, minimizing manual labour by implementing process automation is beneficial **(req. III)**. Fourth, a high level of process control and online monitoring of process parameters should guarantee reproducible production runs **(req. IV)**. Fifth, the reactor system should be designed in the most cost-efficient manner possible **(req. V)**.

In view of these requirements, we developed in this work a completely novel hiPSC-specific suspension culture unit consisting of a customizable CSTR design that is integrated into a fully automated incubator with the possibility for real-time monitoring of aggregate size development during long-term hiPSC suspension culture. To achieve this goal, we utilized a range of engineering tools including computer-aided design (CAD) and computational fluid dynamics (CFD) simulations to enhance the development process. The fabrication of components was achieved by harnessing the flexibility and versatility of 3D printing to construct CSTR parts adapted to the necessary production scale. For convenient handling through system integration, all process control elements and process monitoring data are accessible via one single control system. Further, for the first time, we present a tool that allows for automated visual tracking of hiPSC aggregation in real-time as advanced online process monitoring option. Finally, we demonstrate system functionality by long-term culture of hiPSCs followed by the validation of their pluripotent nature by investigating gene (including PluriTest analysis) and protein expression levels of key pluripotency markers.

## Material and Methods

### CSTR development and fabrication

Miniature CSTR model geometries were generated with the computer-aided design software SolidWorks (Dassault Systèmes, Germany). Glass components were handcrafted by a local glassblower (Glaspunkt, Germany). Impeller and drive shaft geometries were machined from polyether ether ketone (PEEK) and stainless steel, respectively (GT Labortechnik, Germany). Remaining bioreactor components were fabricated from PLA filament by fused deposition modelling (FDM) using an in-house Xeed 3D printer (Leapfrog, Netherlands).

### Computational fluid dynamics

Flow velocity computations were done to mathematically characterize the CSTR fluid regime and calculate the occurring shear stress. For this purpose, finite element method software Comsol 5.3a (Comsol Multiphysics, Germany) was used. Briefly, bioreactor CAD files were imported into Comsol. Model components were meshed and the built-in Reynolds-averaged Navier-Stokes k-ε turbulence model was applied to the bioreactor fluid geometry. Material properties were adjusted as described previously^28^. To improve convergence, viscosity ramping was performed as described in^29^. Following this, the obtained solution of the k-ε turbulence model was utilized as initial condition for a subsequent k-ε low Reynolds re-computation to achieve higher resolution of the viscous buffer layer at the walls for improved shear stress estimation.

### Plasma sterilization of bioreactors

CSTRs were sterilized by vaporized hydrogen peroxide (VH_2_O_2_) plasma sterilization method. First, a plasma chamber (Pico Plasma System, Diener Electronics, Germany) was heated by a 15 min plasma process using a 100 kHz generator and pure oxygen gas (500 W, 0.3 mbar, 12 sccm O_2_). After the first plasma process, the bioreactor lids wrapped in foil (Stericlin, VP Group, Germany) were inserted into the heated chamber, together with a metal vessel containing 1.5 ml of 60% H_2_O_2_ (Thermo Fisher Scientific, USA). In order to vaporize the H_2_O_2_, the chamber was subsequently evacuated to a pressure of 1.0 mbar. The reactors remained in this environment of vaporized H_2_O_2_ for 90 min, followed by a further evacuation step to 0.4 mbar and a second plasma process with the remaining H_2_O_2_ vapour as process gas (4 min, 200 W). Finally, a third plasma process using pure oxygen as process gas was carried out to remove the remaining H_2_O_2_ from the chamber (10 min, 400 W, 12 sccm O_2_).

### hiPSC cell culture

IMR 90-4 (WiCell, USA) monolayer cultures (CTL ML) were maintained on Matrigel®-coated (Corning, Germany) 6-well plates (Nunc, Germany) in mTeSR™1 (StemCell Technologies, Germany) with daily medium change.

As 3D culture of hiPSCs in suspension utilizes mTeSR™3D medium (StemCell Technologies), hiPSC monolayer cultures using Matrigel®-coated plates (Corning) in combination with mTeSR™3D medium were performed as controls (CTL ML 3D). Passaging of CTL ML or CTL ML 3D cultures was done by Accutase® (Sigma-Aldrich, Germany)-treatment followed by re-plating on Matrigel®-coated plates at a 1:3–1:16 ratio in mTeSR™1 or mTeSR™3D supplemented with 10 µM Y-27632 dihydrochloride (Tocris, USA).

For CSTR suspension culture of hiPSCs, first, self-adhesive sensor spots monitoring dissolved oxygen or pH (Presens, Germany) were attached to the inside walls of the CSTR glass vessels. A single-use syringe filter (4.8 cm^2^, 0.2 µm pore size, Sartorius, Germany) was used to allow for gas exchange between the incubator atmosphere and the CSTR headspace. After assembly, CSTRs were sterilized by plasma treatment. hiPSC suspensions were started from monolayer cultures in mTeSR™1. Briefly, mTeSR™1 culture medium was aspirated and cells were washed with phosphate buffered saline (PBS, Thermo Fisher Scientific). Afterwards, PBS was replaced by 1 ml of Gentle Cell Dissociation Reagent (GCDR) per well (StemCell Technologies) and plates were incubated at 37 °C for 5 min. Next, GCDR was replaced by 1 ml of freshly prepared mTeSR™3D seed medium supplemented with 10 µM Y-27632 dihydrochloride followed by detaching hiPSC colonies from the well surface using a cell scraper. Afterwards, cell suspensions were gently passed through a 37 µm cell strainer (StemCell Technologies) to obtain hiPSC colonies of equal size as described by Otsuji and co-workers^30^. Viable cell concentrations were assessed using an automated cell counting system (Nucelocounter NC-200, Chemometec, Denmark). For suspension cultures, CSTRs were inoculated with colonies corresponding to 3.5 × 10^5^ cells per ml in 62.5 ml mTeSR™3D seed medium supplemented with 10 µM Y-27632 dihydrochloride. For further control purposes, 1 ml of the generated hiPSC suspension was cultured as aggregates in standard tissue culture plates without Matrigel® coating using mTeSR™3D medium (CTL SUS). Here, fed-batch cultivation was performed as suggested by the manufacturer. During CTL SUS and CSTR culture, supernatant samples were taken every 24 h for off-line determination of metabolite concentration and lactate dehydroxynase (LDH) activity using a Cedex Bio analyzer (Roche Custom Biotech, Germany). Nutrients and growth factors were replenished for CTL SUS and CSTR cultures by adding mTeSR™3D feed medium for three days following inoculation. For passaging, hiPSC aggregates were dissociated into small clumps on day 4 of each passage. Briefly, cell aggregates were separated from non-viable single cells by filtering aggregate suspensions using a 37 µm reversible strainer. Remaining aggregates were suspended in GCDR for 5 min. After incubation, cells were spun at 100 *g* for 3 min and the supernatant was aspirated. Lastly, the aggregate pellet was resuspended in mTeSR™3D seed medium supplemented with 10 µM Y-27632 dihydrochloride and slowly passed through a 37 µm reversible strainer to obtain hiPSC clumps for re-seeding.

### Real-time imaging of hiPSC aggregates in CSTR cultures

Visual tracking of hiPSC aggregates was realized by circulating hiPSC suspensions through a glass flow chamber located in the field of vision of a custom-built imaging unit (Opto, Germany) inside the incubator. Therefore, cell suspensions were bypassed at 15 ml min^−1^ from and to CSTR vessels using a stationary mounted roller pump (Spetec, Germany). Cell suspensions were circulated for 2 min every 24 h. In-flow images were taken every three seconds and automatically processed at-line by an in-house image analysis algorithm that was developed using ImageJ (ImageJ, USA). From the read-out data, size distribution parameters describing the average aggregate size *µ* and standard deviation *σ* were conveyed. In addition, the number of analysed aggregates per image was extracted to describe the aggregate concentration in each image.

### PluriTest analysis

For PluriTest analysis, RNA was isolated from CTL ML, CTL ML 3D and CSTR samples using the RNeasy® Mini Kit from Qiagen® with an optional on-column DNase Digestion step (RNase-Free DNase Set from Qiagen®, Germany). For microarray analysis, 200–500 ng of total RNA were amplified and biotinylated using the TargetAmp™ - Nano Labeling Kit for Illumina® Expression BeadChip® (Epicentre® - Illumina, USA). Concentration of Biotin-aRNA was measured with a Qubit 3.0 Fluorometer (Thermo Fisher Scientific) and the concentration of each sample was adjusted to 150 ng μl^−1^. 750 ng of Biotin-aRNA were used for hybridization with the HumanHT12v4-Expression BeadChip™ (Illumina, USA) and hybridization was performed at 58 °C for 16–20 h. After hybridization, BeadChips were washed and stained according to the manufacturer’s standard protocol. BeadChips were scanned using the iScan instrument from Illumina®. Raw data (*.idat files) were submitted to PluriTest analysis (www.pluritest.org). PluriTest results were normalized using a published script^31^.

### Flow cytometry

Single-cell suspensions of hiPSCs were prepared by Accutase®-treatment. All steps were performed at 4 °C. In total, 2 × 10^5^ cells were stained per sample. Centrifugation steps were performed at 250 *g* and 4 °C. Cells were washed two times in 1X BD Perm/Wash™ buffer (BD Biosciences) prior to 20 min of incubation in PERM/FIX (BD Biosciences) solution for permeabilization and fixation of cells followed by another washing step and primary antibody incubation. For analysis of Oct4, Sox2 and Nanog, cells were incubated for 30 min at 4 °C with PE mouse anti-Oct3/4 (1:50; Human Isoform A, BD Biosciences), rat anti-Sox2 (1:50; Alexa Fluor® 488 Conjugate, eBioscience, USA) and Nanog (D73G4) XP® Rabbit mAb (1:50; Alexa Fluor® 647 Conjugate, Cell Signaling Technology, USA). Corresponding isotype controls were incubated with PE mouse IgG1 kappa isotype control (1:50; BD Biosciences), rat IgG2a kappa isotype control Alexa Fluor® 488 Conjugate (1:50; eBioscience) and Rabbit (DA1E) mAb IgG XP® Alexa Fluor® Conjugate 647 (1:100; Cell Signaling Technology). Afterwards, cells were washed two times in 1X BD Perm/Wash™ buffer followed by one washing step in FACS buffer (PBS^−^, 1% FCS, 2 mM EDTA (Sigma-Aldrich). Cells were analysed using a BD FACS Accuri C6 plus flow cytometer (BD Biosciences).

### Quantitative RT-PCR

RNA isolation was performed using the RNeasy Micro or Mini Kit (Qiagen, Germany). cDNA was synthesized using the iScript^™^ cDNA Synthesis Kit (Biorad, Germany). qRT-PCR was performed on the CFX96 Touch^™^ Real-Time PCR Detection System (Biorad) using SsoFast^™^ EvaGreen^®^ Supermix (Biorad). For calculation of gene expression, the 2^−ΔΔCt^ method was used with *hRPL6* and *hRPL4* as reference genes. Reactions were performed in duplicates at 60 °C annealing temperature. The following primer sequences were used: *hRPL6* (FW: 5′-ATTCCCGATCTGCCATGTATTC-3′, REV: 5′-TACCGCCGTTCTTGTCACC-3′), *hRPL4* (FW: 5′-GCCTGCTGTATTCAAGGCTC-3′, REV: 5′-GGTTGGTGCAAACATTCGGC-3′), *hOCT4* (FW: 5′-CCTCACTTCACTGCACTGTA-3′, REV: 5′-CAGGTTTTCTTTCCCTAGCT-3′), *hSOX*2 (FW: 5′-CCCAGCAGACTTCACATGT-3′, REV: 5′-CCTCCCATTTCCCTCGTTTT-3′), and *hNANOG* (FW: 5′-CCAAAT TCTCCTGCCAGTGAC-3′, REV: 5′-CACGTGGTTTCCAAACAAGAAA-3′).

### Statistical analysis

Statistical analysis was performed using GraphPad Prism 6. All quantitative data were investigated for statistical deviations using a one-way ANOVA employing Dunnett’s test. A *p*-value < 0.05 was considered significant with *n* = 3 for all experiments.

## Results

### Conceptual design of a 3D suspension culture unit specific for hiPSCs through *in silico* analysis and additive manufacturing

**Figure 1:**
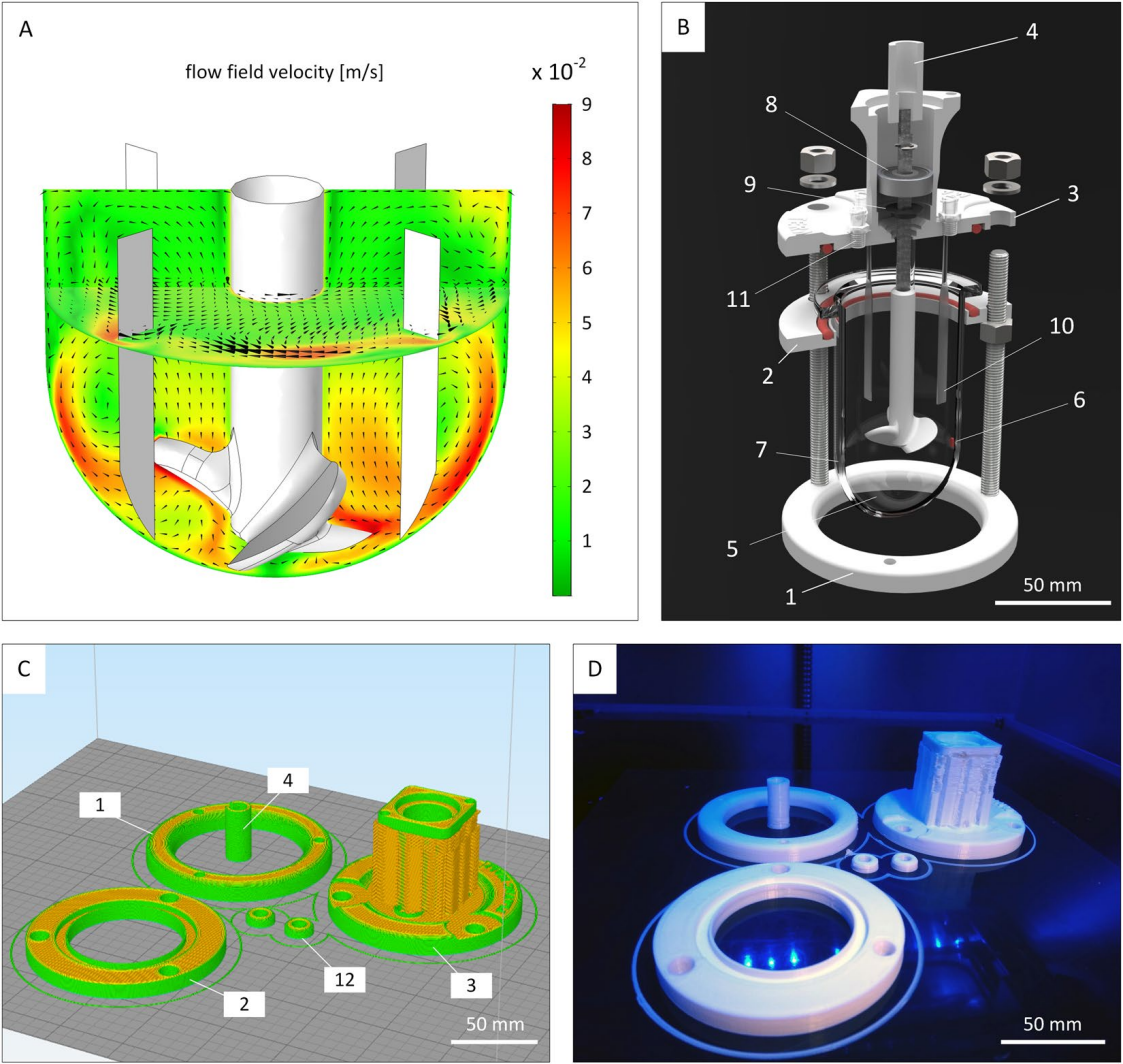
Bioreactor design and fabrication. **(A)** Computational fluid dynamic software was used to identify an appropriate impeller configuration and fluid flow regimes for human induced pluripotent stem cell cultivation *in silico*. **(B)** The custom-made miniature continuously stirred tank reactors (CSTRs) were designed for operation in an incubator. The largest components are the baseplate (1), the glass support (2), the lid (3), and the motor coupling (4). These parts are enclosing the culture glass vessel (5) and were produced by in-house fused filament fabrication (3D printing). All reactors are equipped with optical oxygen sensor spots (6) and pH sensor spots (7) that are attached to the inside of the glass wall. The rotating shaft is stabilized by a stainless steel ball-bearing (8). Sterile conditions inside the vessel are ensured by a shaft seal (9) that is placed below the ball-bearing. A total of four baffles (10) promote homogenous fluid mixing. Luer-lock ports (11) provide head-space gas exchange with the surrounding atmosphere of the incubator through air filters and also allow fluid removal and fluid addition by riser pipes. **(C)** Computer-aided design sketches of bioreactor components at the beginning of the manufacturing process. Components are translated into mesh files that are subsequently sent to the 3D printing software that automatically outlines the print flow. Component labelling is identical to (**B**). In addition, caps for riser pipe ports (12, not shown in (**B**)) are added to the printing interface. **(D)** Components at final stage of printing after approximately 4 h.

The aim of this study was to construct a cell culture unit consisting of a custom-made incubator and a newly designed hiPSC-specific CSTR that allows real-time monitoring of hiPSC aggregation. To meet the defined requirements as mentioned above **(req. I–V)**, a variety of engineering tools and methods were utilized, e.g. computer-aided design, computational fluid dynamics, and additive manufacturing. The first objective was to generate a CSTR that would avoid massing and subsequent clumping of settled aggregates due to poor mixing. Therefore, in a first step, a suitable vessel shape and impeller design that leads to thorough uplift flow was identified. In this context, we evaluated the fluid flow regime of various small-scale bioreactor designs by running computational fluid dynamics simulations. As a result, we successfully derived a baffled agitation setup that generates adequate flow velocities at the bottom of the vessel to keep hiPSC aggregates in suspension and that prevents cell clumping (Fig. 1A). Moreover, shear stress was kept at 5.9 × 10^−2^ dyne cm^−2^ in average when agitating at 75 revolutions per minute (RPM). Following computational fluid dynamics simulations of the vessel shape, a full bioreactor design was created *in silico* using CAD software. Here, efforts were made to minimize the total number of components for improved CSTR handling (Fig. 1B). To minimize costs, most parts were subsequently produced from PLA by using an in-house 3D printer. The fabrication process of a single bioreactor was accomplished within 4 h (Fig. 1C,D, Supplementary Video 1).

**Figure 2:**
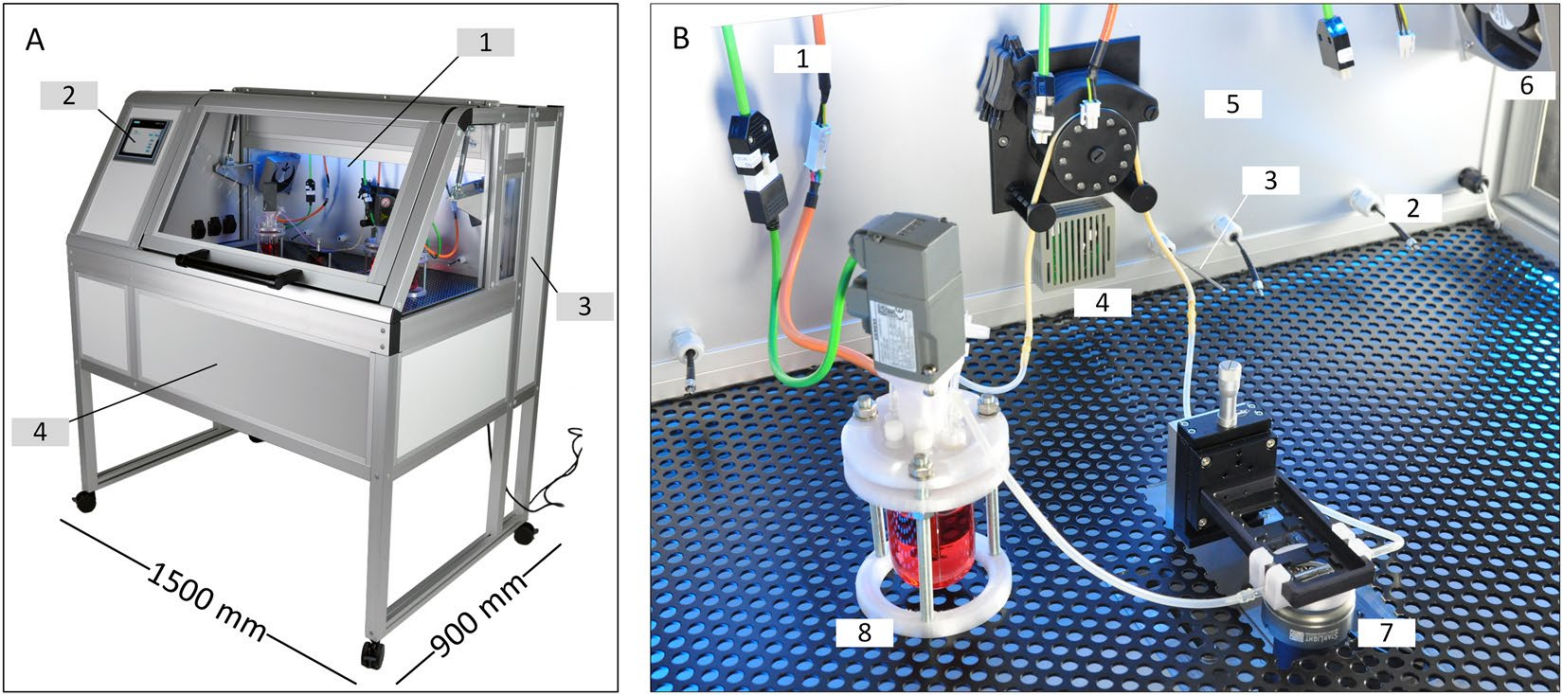
Hardware setup of cell expansion platform. **(A)** A tailor-made incubator system was constructed to provide appropriate culture conditions for human induced pluripotent stem cell suspensions. The heart of the incubator is the incubated space (1) that accommodates the suspension culture vessels. The incubator atmosphere control, liquid-handling device settings, impeller agitation speed and data management is adjustable via human machine interface (2). The process control equipment and sensor transmitters are situated at the backside compartment of the incubator (3). The *in situ* imaging microscope is incorporated into the lower compartment (4). **(B)** The incubator was designed to operate multiple miniature CSTRs in parallel. Therefore, motor power cables are available to run three reactors simultaneously (1). Equally, optical oxygen measurement and pH measurement is available for each reactor in operation (2). The incubator atmosphere was set to 37 °C and 5% CO_2_ with the aid of temperature (3) and carbon dioxide (4) sensors. The incubation chamber was tempered by heating foils attached to the rear panel of the incubator (5). Fans (6) to either side of the incubation chamber ensured constant air circulation. Real-time imaging of cell aggregates was achieved by passing a portion of the cell suspension from the reactors across a fixed microscope (7). CSTRs (8) were placed freely inside the incubator.

For housing the CSTRs and measuring equipment, a custom-made incubator was designed and constructed, enabling the operation of up to three CSTRs simultaneously (Fig. 2A). To guarantee standard cell culture conditions, CO_2_ and temperature sensors were installed to support regulation of the incubation atmosphere via Programmable Logic Controller (PLC) system (Fig. 2B). To allow for pH and dissolved oxygen measurements of CSTR cultures, the incubator was equipped with hardware for optical sensor spot-based pH and dissolved oxygen sensing. Therefore, fibre cables were run from the incubation space to the required transmitters that were installed in the rear cabinet of the incubator. Further on, the incubator was equipped with a peristaltic pump that facilitated automated liquid handling. In this study, the pump was used to transport hiPSCs in suspension from the CSTR to the built-in *in situ* microscope in the centre of the incubation space during in-flow hiPSC aggregate imaging. The incubator PLC system-based operation was managed via a single human-machine interface (HMI) that was installed in the incubator front panel. The HMI allowed the user to set the incubator atmosphere composition; to log process parameter data; to access the automated liquid handling for *in situ* imaging; and to set the CSTR agitation speed.

### Real-time monitoring of hiPSC aggregation during suspension culture in CSTRs

**Figure 3:**
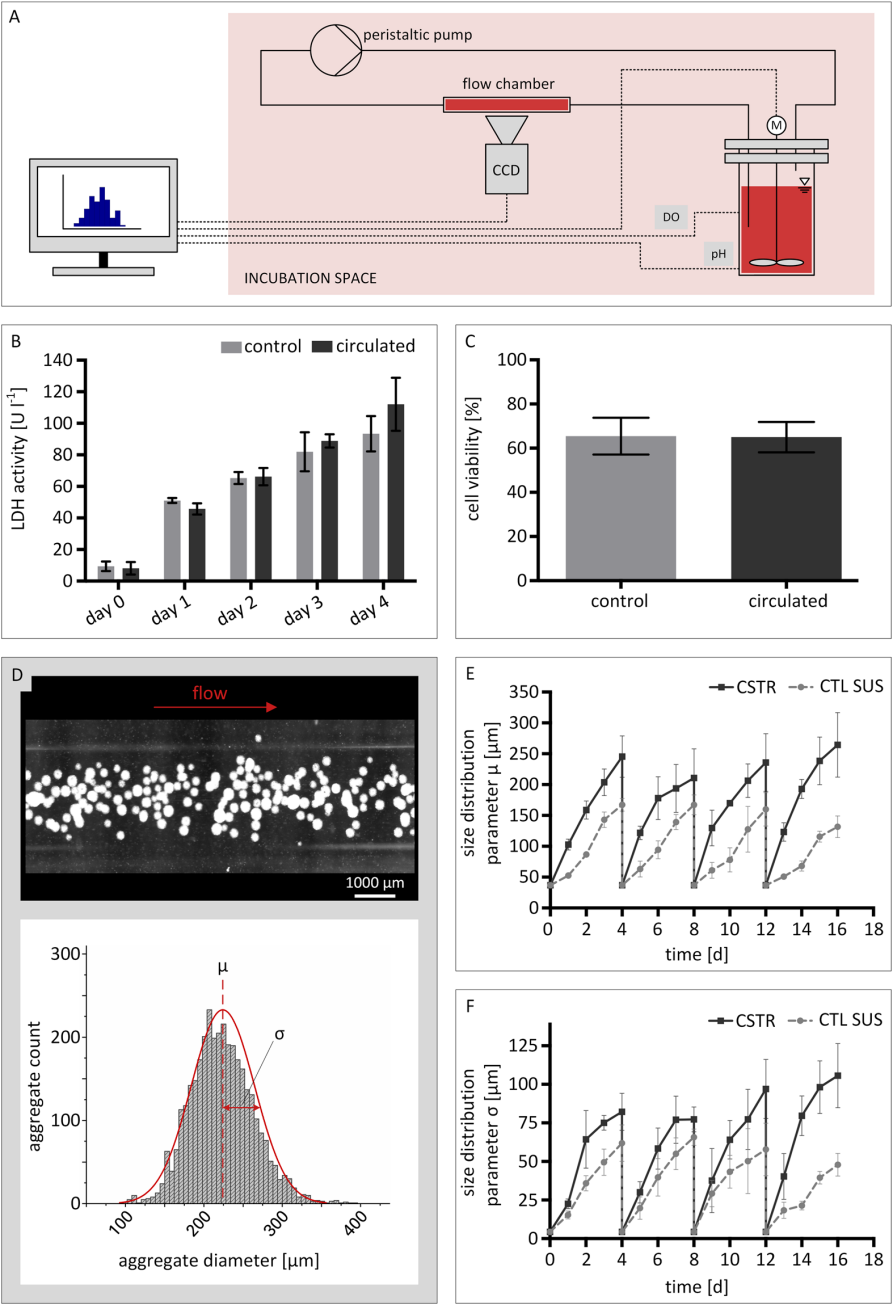
Automated real-time imaging of human induced pluripotent stem cell suspension cultures. **(A)** Outline of the cell suspension circulation. Cell suspension was sampled from the reactor by peristaltic pumping and led through a flow chamber above the microscope lens. Images were acquired via CCD camera while the suspension is in flow. A computer was used for at-line image processing, impeller speed control, and pH and oxygen measurement recording. **(B)** Suspension culture samples were investigated for lactate dehydroxynase activity and **(C)** cell viability to convey potential cell damage caused by the peristaltic pump during circulation. **(D)** Processing of suspension culture images provided aggregate size distributions. Here, the mean aggregate diameter *µ* and its standard deviation *σ* are used as numerical readout parameters. Automated real-time imaging allowed for detailed characterization of the aggregate formation during bioreactor culture by plotting **(E)** the average aggregate diameter *µ* and **(F)** the standard deviation *σ* of the aggregate diameter *µ* as a function of cultivation time. Error bars in all subfigures indicate mean ± SD from *n* = 3 experiments.

In order to realize real-time monitoring of hiPSC aggregate formation in CSTR suspension cultures, a liquid handling bypass was connected to each bioreactor (Fig. 3A). Prior to bioreactor experiments, preliminary studies were performed to exclude potential harm inflicted on hiPSC aggregates during automated liquid handling. For this purpose, small-scale static hiPSC suspension cultures of 10 ml were circulated every 24 h for 4 days. To assess cell damage, lactate dehydrogenase (LDH) activity (Fig. 3B) was measured on a daily basis. LDH is an oxidoreductase enzyme that is, when secreted by cells, commonly associated to cell damage and loss of cell integrity. In addition, cell viability was determined on day 4 of culture (Fig. 3C). All measurements were compared to static control suspension cultures (CTL SUS). As a result, no variation in LDH activity was detected at any time point. Similarly, no alteration between the circulated sample and the control was found for cell viability.

**Figure 4:**
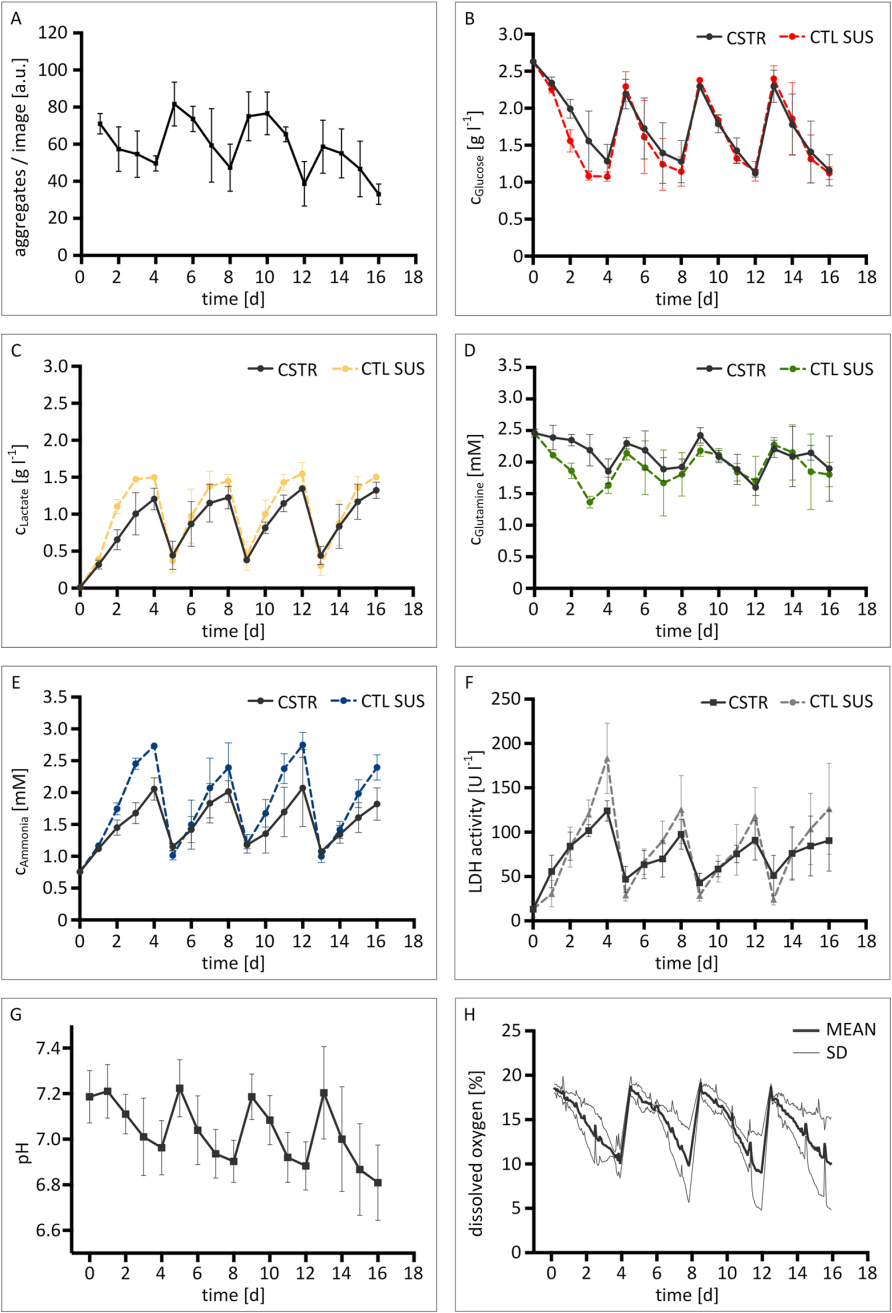
Metabolite concentrations and process parameter profiles of hiPSC suspension cultures in bioreactors and static well-plate suspension control cultures. *In situ* imaging facilitated the evaluation of aggregate concentrations on in-flow images **(A)**. At-line analysis of culture medium supernatant allowed for metabolite concentration assessment of **(B)** glucose, **(C)** lactose, **(D)** glutamine, **(E)** ammonia, **(F)** and lactate dehydrogenase activity. Bioreactors were equipped with optical sensor spots for measuring physical process parameters **(G)** pH and **(H)** dissolved oxygen. Error bars indicate mean ± SD, *n* = 3.

Next, real-time monitoring of hiPSC aggregation in the developed CSTRs was conducted by using the built-in custom-made *in situ* microscope. In total, 40 images were taken within one session, providing approximately 2000 aggregate counts per measurement within 2 min. An exemplary image taken by *in situ* microscopy is shown in Fig. 3D. In addition, images of CTL SUS controls were taken using a digital lab microscope (EVOS XL, Thermo Fisher Scientific). In this case, a total of approximately 100 counts were achieved per image. The process of manual imaging was accomplished within 1–2 min on average. From all images, statistical parameters linked to aggregate size distribution were derived. Hereby, the mean aggregate diameter *µ* together with the mean diameter’s standard deviation *σ* were identified as suitable parameters to characterize forming aggregates (Fig. 3D, bottom). Additionally, the aggregate concentration in each image was determined (Fig. 4A).

Plotting of the mean aggregate diameter *µ* revealed recurring size increases throughout each passage (Fig. 3E). At the same time, the concentration of aggregates per in-flow image was observed to decrease (Fig. 4A). For CSTR and static CTL SUS cultures, the starting size of aggregates in every passage was observed to be approximately 50 µm. From there on, aggregate size development was detected to vary between culture formats. For CSTR cultures, mean aggregate diameters of approximately 120 µm were detected 24 h after re-seeding. Towards the end of each passage, mean aggregate diameters were no greater than 260 µm. Static CTL SUS cultures, on the other hand, showed lower aggregate diameters from day 1 onwards. Here, mean aggregate diameters were consistently smaller by 50–90 µm compared to CSTR cultured aggregates. In accord to this observation, end-point aggregate sizes increased to about 150 µm.

For in-flow imaging of CSTR cultures, the concentration of aggregates in each image was determined. Here, 72 aggregates in average were observed on each in-flow image 24 h after re-seeding. End-point measurements revealed considerably lower aggregate numbers of approximately 40 counts per image towards the end of each passage (Fig. 4A).

To assess aggregate size uniformity, the mean diameter’s standard deviation *σ* was investigated as an additional size distribution parameter (Fig. 3F). Starting at approximately 5 µm for CTL SUS and CSTR setups, the average standard deviation *σ* increased to 75 µm for CSTR cultures during passage 1 and 2. However, throughout the third and fourth passage, notably higher deviations were detected. Here, the standard deviation resulted in end-point measurements in the range of 100 µm. Static control cultures showed comparably lower end-point deviation values of approximately 60 µm during all passages.

### CSTR-cultured hiPSCs are healthy and vital

Growing and maintaining hiPSCs in uncommon cell culture systems is often linked to cellular toxicity. To this aim, we assessed the metabolic activity of hiPSC CSTR and CTL SUS cultures by at-line analysis of diverse metabolites within the culture medium to investigate hiPSC health status.

Recurring metabolite concentration patterns for glucose, lactate, glutamine, and ammonia were detected, indicating functional carbon metabolism throughout all passages. The concentration of carbon sources glucose and glutamine was observed to never drop below 1 g l^−1^ and 1.2 mM, respectively, implying sufficient energy sources in both CSTR and CTL SUS culture formats (Fig. 4B,D). For the corresponding waste products, lactate and ammonia peak concentrations of approximately 1.5 g l^−1^ and 2.5 mM were detected (Fig. 4C,E). The concentration of lactate in CTL SUS thereby slightly surpasses the critical level of 1.3 g l^−1^ (15 mM) that has been reported to impair cell proliferation and productivity in hiPSCs^32^.

In addition, we determined LDH activity to investigate physical cell impairment. As shown in Fig. 4F, comparable levels of LDH activity were detected for CSTR cultures and CTL SUS controls. However, LDH activity was stronger in CSTR cultures shortly after passaging. Additionally, end-point LDH activity was increased in CTL SUS compared to CSTR cultures. Further, we measured dissolved oxygen content and pH levels by using optical sensor spots during culture of hiPSCs in the developed CSTRs. In this case, pH values were in the range of 6.8 at their lowest, and approximately 7.2 immediately after passaging (Fig. 4G). Dissolved oxygen levels remained above anoxia at all times and were restored to ambient oxygen content of 20% through medium change on passaging days (Fig. 4H).

### hiPSCs retain pluripotent when cultured in CSTRs

**Figure 5:**
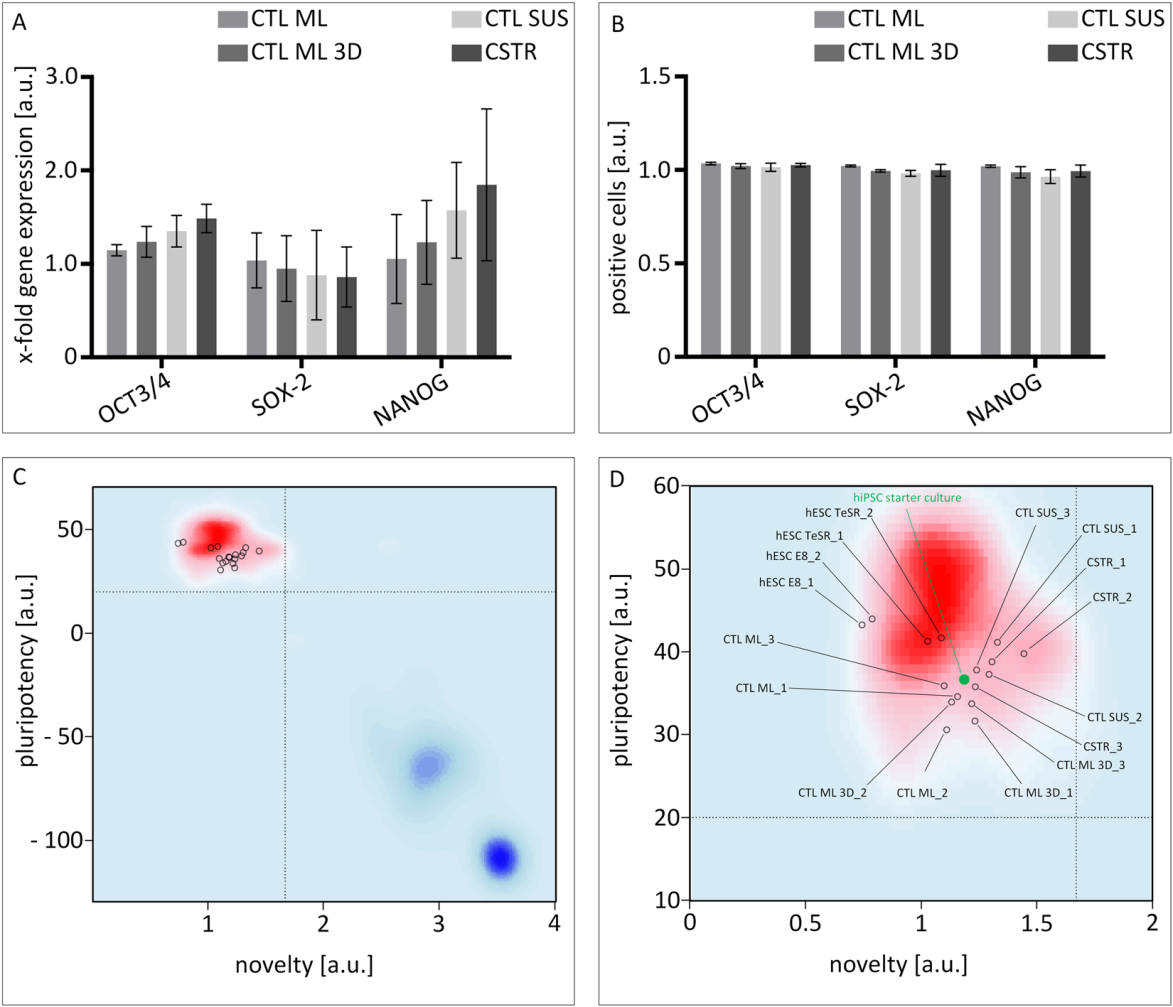
hiPSC preserve pluripotency throughout four passages. Cells grown as monolayer culture (ML) and suspension culture (SUS) in either mTeSR™1 or mTeSR™3D cell culture medium were investigated for pluripotency status on gene and protein level. **(A)** The gene expression of pluripotency-associated markers *OCT3/4*, *SOX-2*, and *NANOG* was confirmed by qRT-PCR. **(B)** Likewise, flow cytometry analysis revealed consistent expression of pluripotency transcription factors. Values in (A) and (B) are normalized to marker expression of pluripotent starting cultures. Error bars indicate mean ± SD, *n* = 3. **(C)** PluriTest results (HT12v4 arrays) normalized to hESC H9 cells cultured in E8 and TeSR medium (*n* = *2* for normalization samples). The background encodes an empirical density map indicating areas of high Pluripotency/low Novelty Scores (red) and high Novelty/low Pluripotency Scores (blue); thresholds for Pluripotency (20, horizontal) and Novelty (1.67, vertical) Scores are indicated with dashed lines. All samples pass both Pluripotency and Novelty Score criteria. **(D)** Magnification of PluriTest results from (**C**) hiPSC starter culture is highlighted green.

Changing cell culture conditions for pluripotent stem cells is often associated with impaired cell proliferation, the loss of pluripotency, and spontaneous differentiation. In this study, cell concentration measurements revealed a 2.75 ± 0.84-fold expansion of hiPSCs in stirred suspension vessels per passage, and aggregates showed normal morphology (Fig. S1). To provide evidence for pluripotent stem cell qualities using the developed CSTRs, we first analysed the expression of key pluripotency markers Oct4, Sox2 and Nanog^33^ after 20 days in CSTR culture by qRT-PCR and flow cytometry. As shown in Fig. 5A, CSTR hiPSC cultures showed similar gene expression patterns for *OCT4*, *SOX2* and *NANOG* compared to standard CTL ML controls. Further, gene expression profiles of the CTL ML 3D and CTL SUS controls were also comparable. In addition, flow cytometry data revealed robust protein expression of OCT4, SOX2 and NANOG in CSTR-hiPSCs. Similar levels were obtained for CTL ML, CTL ML 3D and CTL SUS-cultured hiPSCs, indicating that the developed CSTR system represents a suitable culture format for maintaining the pluripotent state of hiPSCs in suspension (Fig. 5B). We further assessed pluripotency characteristics of hiPSCs in the different culture conditions using an unbiased bioinformatics assay termed PluriTest^34^. Here, a global transcriptomic assessment of pluripotency is achieved by computing transcriptomic raw data (query) against an empirical model generated from hundreds of pluripotent and non-pluripotent cells and tissues. Output characteristics of the PluriTest algorithm are Pluripotency and Novelty Scores. If criteria for both scores are met, the cells can be considered as pluripotent. A summary of the data is shown in Fig. 5C,D, and all samples pass both Pluripotency and Novelty Score criteria.

## Discussion

To overcome the limitations of standard suspension culture of hiPSCs, we defined a set of requirements (req. I-V) that were subsequently fulfilled with the help of a variety of engineering tools. The prime requirement was to expand high quality stem cells exhibiting a pluripotent nature in a most robust fashion **(req. I)**. In this regard, a 2.75-fold expansion of hiPSC was observed in average, and hiPSC identity was demonstrated by the expression of the key pluripotency markers Oct4, Sox2, and Nanog as well as by PluriTest analysis (Fig. 5C,D).

We further demonstrate the feasibility of the suspension culture platform for one specific CSTR model. Yet, in order to address the requirement of high scaling flexibility, we utilized a combination of computer-aided design, computational fluid dynamics, and fused deposition modelling to lay the foundation for future bioreactor systems of adjustable scale that are compatible with CSTR-based culture formats for hiPSC culture **(req. II)**. A major advantage of using 3D printed components lies in the flexibility of cultivation vessel design and scale. Here, the vessel size is easily adjustable to the required level of scaling and the fabrication process is completed within a few hours (Fig. 1C,D). At the same time, the platform concept allows for extra scaling flexibility as the measurement equipment and drive connection is independent from the vessel size, and the incubator is capable of operating CSTRs of various working volumes simultaneously. An exemplary three-CSTRs setup is shown in the supplementary (Fig. S2).

Another strength of utilizing a platform concept for hiPSC suspension cultures lies in the option to implement process control, and thereby reduce labour-intensive manual handling steps **(req. III)**. For an example, a peristaltic pump was used for automated sampling of hiPSC suspension for subsequent *in situ* imaging (Fig. 3A). However, the custom-built incubator is capable of performing additional automated liquid handling steps in future experiments, e.g. the automated addition of feed medium to CSTR cultures and harvest of cell suspensions on passaging days.

Throughout the culture duration, the custom-built incubator facilitated continuous monitoring of the incubation periphery and bioreactor state parameters, e.g. pH, dissolved oxygen, temperature, and CO_2_. In addition, the development of aggregation was successfully tracked and thus supported reproducible cell expansion cycles **(req. IV)**. By exploiting an automated flow-chamber-approach, we provide a monitoring tool capable of characterizing the size distribution of forming hiPSC aggregates at high precision. Referring to the obtained data, we show that our hiPSC culture vessels lead to the steady formation of aggregates at recurring size patterns that are confined to the used suspension culture format (Fig. 3E). We further show that the suspension culture platform is able to generate data on aggregate formation at a rate of approximately 1000 aggregates per minute. In contrast to manual sampling and imaging procedures where considerably lower counts are analysed^35,36^, our technology delivers data with statistical relevance while omitting cell loss due to sampling, an important aspect in stem cell propagation.

Size distribution is considered a process parameter that is confined to aggregate-forming species. On the one hand, monitoring of aggregate sizing is vital to monitor the potential formation of oversized aggregates that are prone to starvation and/or undirected lineage commitment in regions close to the aggregate core. More importantly, our findings confirm the postulation of aggregate development being highly dependent on the suspension culture vessel and the agitation setup^37^. This is exemplified when comparing aggregate size development in CSTR cultures and static cultures (Fig. 3E). Deviations between culture formats are largely caused by the increased probability of aggregates to merge into larger species when the cell suspension is actively agitated. This aspect is further elucidated by the decreasing concentration of hiPSC aggregates towards the end of each passage, indicating the steady coalescence of readily formed smaller hiPSC aggregates (Fig. 4A). The overall aggregation process, however, is further dependent on multiple factors such as seeding density, impeller design, and RPM.

Since the newly developed real-time imaging device is based on a suspension bypass, it is independent from culture vessel dimensions and is thus applicable for future scaling studies in bioreactors of various sizes. Lastly, a detailed characterization of aggregate size development is essential to interpret energy metabolism and cell health in aggregate cultures. For instance, the activity of LDH enzymes was observed to be consequently higher in CSTR cultures at the beginning of each passage, indicating that cells are more prone to lysis in agitated environments after re-seeding (Fig. 4F). In the end, however, LDH activity was greater in CTL SUS cultures towards passaging days. Considering the observation that hiPSC self-aggregation is enhanced during CSTR culture, the data consequently suggest that aggregation protects hiPSC from cell lysis. The analysis of metabolites in CSTR and CTL SUS cultures further indicate that cell proliferation is increased in non-agitated culture setups. This is demonstrated by the comparably rapid accumulation of ammonia and lactate in the culture medium and, as a consequence, higher waste product concentrations at the end of each passage (Fig. 4C,E). Again, if the aggregate size is taken into account, the results show that energy metabolism is weaker in larger aggregates, probably due to diffusional limitations and commencing oxygen limitation in cells close to aggregate cores^38^.

As a matter of fact, the main financial expenses in hiPSC culture arise from the necessity for suitable matrices like Matrigel®, cell culture media, and bioreactor equipment. Still, in this study several entry points for cost reduction were exploited. With the help of engineering tools including computer-aided design, computational fluid dynamics simulations, and 3D printing, the development of new bioreactors was readily accomplished and prototype production time and costs were greatly reduced. Initially, computational modelling was used to characterize a variety of design approaches before entering the prototype production phase (data not shown). Here, a shape combination of vessel and impeller that allows reliable suspension of hiPSC aggregates through thorough mixing while keeping shear forces at acceptable levels was identified (Fig. 1A). In this context, an average shear stress of 5.9 × 10^−2^ dyne cm^−2^ was calculated for the final CSTR design during operation. Since the conveyed shearing is below shear stress reported to enhance mechanically-induced germ layer differentiation^39,40^, the bioreactor model components were subsequently transferred to the production stage. Besides few components e.g. the bioreactor glass vessel, the agitation shaft with ball bearing (Fig. 1B), larger bioreactor components were fabricated by in-house 3D printing (Fig. 1C). The impeller was machined from PEEK as the printing resolution of the 3D printer was insufficient to meet the amount of detail that was required for the impeller blade curvatures. However, stereolithography (SLA) 3D printing strategies that allow high-resolution printed components are feasible. Besides the above-mentioned advantage in scaling flexibility, the use of 3D printing drastically decreases overall material costs. For printable materials such as PLA, the material expenses are negligible compared material prices of commonly used materials like stainless steel or PEEK. Due to the retrenchments, we were able to run fully monitored hiPSC cultivations at estimated hardware costs that are approximately 75% lower compared to commercially available cultivation systems **(req. V)**.

In the current CSTR design, handcrafted glass vessels were used that ultimately dictated the overall bioreactor scale at this state. However, in the future, full bioreactor models that are almost fully derived from 3D printers are feasible. In this context, alternative high-resolution SLA 3D printing strategies are currently under development. As part of novel printing strategies, material tests become necessary to extent the data on material cytotoxicity performed for this study (Fig. S3). Thereby, the proportion of non-printed hardware parts such as glass components can be further minimised while scaling flexibility is escalated.

In a similar manner, further module additions to the suspension culture platform are conceivable. For instance, a refrigerated storage cabinet for cell culture media and feed medium batches is going to allow for additional liquid handling options to further reduce manual labour. The *in situ* microscope was custom-made for the incubator setup and is especially designed for the assessment of aggregate sizes through dark field imaging. To further enhance the visual monitoring of hiPSC suspension cultures, the installation of a fluorescence microscopy module is possible. By doing so, automated assessment of a variety of hiPSC attributes such as differentiation progress becomes possible.

## Conclusion

To face the increasing demand for large numbers of high-quality stem cells, we utilized a variety of engineering tools to develop and construct a highly versatile and automated, CSTR-based cell culture unit for scalable hiPSC expansion in suspension. We validated the functionality of our system by confirming the maintenance of the pluripotent cell state on single and global transcript level (PluriTest), and on protein level. At this state, the suspension culture unit facilitates monitoring of hiPSC aggregation at statistically relevant accuracy and in real-time while keeping manual handling efforts and material costs at a minimum.

### Supplementary information

Supplementary information

Supplementary Video 1

Supplementary Video 2

## Data Availability

The datasets generated during and/or analysed during the current study are available from the corresponding author on reasonable request.
