# Author Correction: Automated real-time monitoring of human pluripotent stem cell aggregation in stirred tank reactors

**DOI:** 10.1038/s41598-024-57467-3

**Published:** 2024-04-02

**Authors:** Ivo Schwedhelm, Daniela Zdzieblo, Antje Appelt-Menzel, Constantin Berger, Tobias Schmitz, Bernhard Schuldt, Andre Franke, Franz-Josef Müller, Ole Pless, Thomas Schwarz, Philipp Wiedemann, Heike Walles, Jan Hansmann

**Affiliations:** 1 University Hospital Würzburg, Department Tissue Engineering and Regenerative Medicine (TERM), 97070 Würzburg, Germany; 2 Translational Center for Regenerative Therapies, Fraunhofer Institute for Silicate Research ISC, 97070 Würzburg, Germany; 3 University Hospital Schleswig-Holstein, Department of Psychiatry and Psychotherapy, 24105 Kiel, Germany; 4 University Hospital Schleswig-Holstein, Institute of Clinical Molecular Biology, 24105 Kiel, Germany; 5 Fraunhofer Institute for Molecular Biology and Applied Ecology IME, 22525 Hamburg, Germany; 6 Mannheim University of Applied Sciences, Institute of Molecular and Cell Biology, 68163 Mannheim, Germany

Correction to: *Scientific Reports* 10.1038/s41598-019-48814-w, published online 23 August 2019

This Article contains an error.

In the Materials and Methods section, the automated cell counter ‘NucleoCounter®’ and the manufacturer ‘ChemoMetec’ were incorrectly given as ‘Nucelocounter’ and ‘Chemometec’, respectively.

“Viable cell concentrations were assessed using an automated cell counting system (Nucelocounter NC-200, Chemometec, Denmark).”

should read,

“Viable cell concentrations were assessed using an automated cell counting system (NucleoCounter® NC-200^TM^, ChemoMetec, Denmark).”

