# Supplementary material for: Automated real-time monitoring of human pluripotent stem cell aggregation in stirred tank reactors
Source: Sci Rep. 2019 Aug 23;9:12297. doi: 10.1038/s41598-019-48814-w (PMC6707254; doi:10.1038/s41598-019-48814-w)
Supplement: Supplementary file 1 — Supplementary information [file 41598_2019_48814_MOESM1_ESM.pdf]

Supplementary information: Automated real-time monitoring of human pluripotent stem cell aggregation in stirred tank reactors

Ivo Schwedhelm[1], Daniela Zdzieblo[1], Antje Appelt-Menzel[1,2], Constantin Berger[1], Tobias Schmitz[1], Bernhard Schuldt[3], Andre Franke[4], Franz-Josef Müller[3], Ole Pless[5], Thomas Schwarz[2], Philipp Wiedemann[6], Heike Walles[1,2], Jan Hansmann[1,2]*

[1]University Hospital Würzburg, Department Tissue Engineering and Regenerative Medicine (TERM), 97070 Würzburg, Germany

[2]Translational Center for Regenerative Therapies, Fraunhofer Institute for Silicate Research ISC, 97070 Würzburg, Germany

[3]University Hospital Schleswig-Holstein, Department of Psychiatry and Psychotherapy, 24105 Kiel, Germany

[4]University Hospital Schleswig-Holstein, Institute of Clinical Molecular Biology, 24105 Kiel, Germany

[5]Fraunhofer Institute for Molecular Biology and Applied Ecology IME, 22525 Hamburg, Germany

[6]Mannheim University of Applied Sciences, Institute of Molecular and Cell Biology, 68163 Mannheim, Germany

Corresponding author:

Prof. Dr.-Ing. Jan Hansmann, University Hospital Würzburg, Department Tissue Engineering and Regenerative Medicine

Jan.Hansmann@uni-wuerzburg.de

Röntgenring 11, 97070 Würzburg

Phone +49 931 31-81209 | Fax +49 931 31-81068

Cytotoxicity assay method

When opting for material alternatives for bioreactor construction, several choices exist beyond the stainless steel (S/S) gold standard: high performance plastics such as polyether ether ketone (PEEK) or polyoxymethylene (POM), and budget materials used in fused deposit modelling 3D printers like polylactic acid (PLA). Besides the glass vessel, the cell suspension is in direct contact with the agitating impeller material only. Nonetheless, the suspension headspace is enclosed by the reactor lid, which is commonly considered as a direct connection through condensation of the liquid and thus requires material cytotoxicity assessment. To investigate the impact of possible material leaching on hiPSC viability, ATP-content-based cytotoxicity tests were performed. Therefore, a sample of each material was sterilized by plasma treatment and soaked in 10 ml of freshly prepared mTeSR™3D seed medium supplemented with 10 µM Y-27632 dihydrochloride at 37 °C for 24 h. After incubation, material samples and incubation medium were asceptically separated. The incubated medium was then used to start hiPSC static suspension cultures of 1 ml Nunclon™Delta multi-well plates (Thermo Fisher Scientific) at 3 x 10^5 cells ml^{-1}. Subsequently, suspension cultures were incubated at 37 °C for 24 h in standard cell culture incubators. Potentially cytotoxic effects were assessed by CellTiter GLO® luminescence (Promega, Germany) ATP measurements following the manufacturer's instructions. Triplicates of samples were measured using an Infinite M200 microplate reader (Tecan, Germany).

Supplementary figures

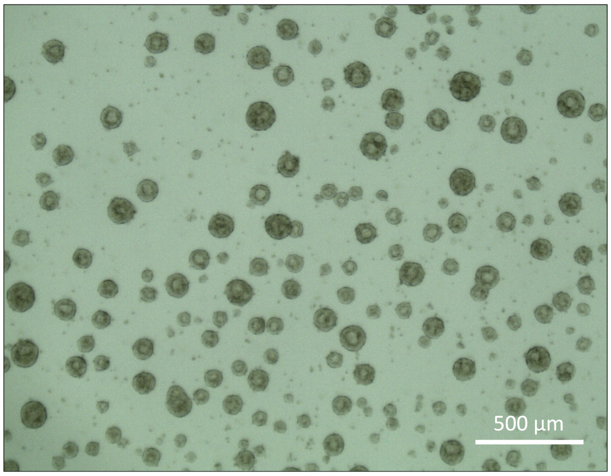

Fig. S1: CSTR-cultured hiPSC aggregates showed normal morphology throughout CSTR culture. Example image of aggregates sampled from a CSTR on day 3 of passage 4.

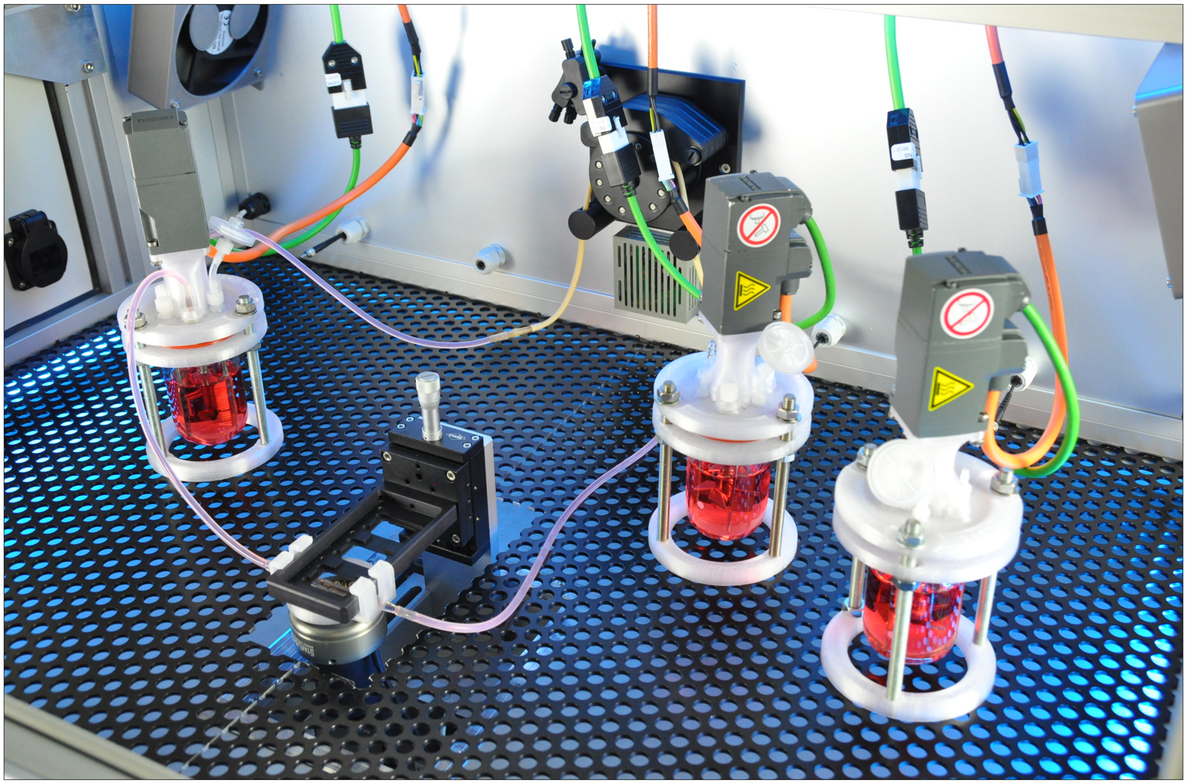

Fig. S2: Demonstration of the tailored incubator's bioreactor capacity. The developed suspension culture platform offers space and monitoring equipment for running three bioreactors in parallel. For clarity, only one CSTR is connected to the *in situ* imaging unit in the center of the incubator.

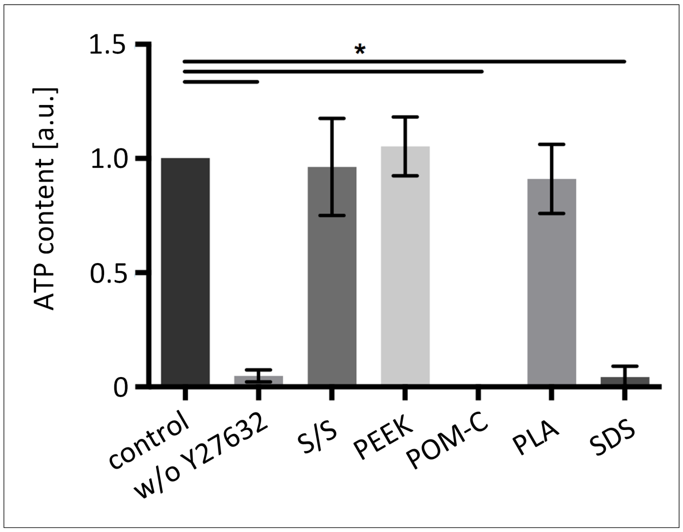

Fig. S3: Cytotoxicity testing of potential bioreactor construction materials. Stem cell culture medium was incubated with sterile material samples made of stainless steel (S/S), polyether ether ketone (PEEK), polyoxymethylene (POM), and polylactic acid (PLA). The incubated medium was inoculated with hiPSCs and cell viability was determined to indicate cytotoxic effects caused by material leaching. Negative controls comprised of hiPSC inoculated in sodium dodecyl sulfate (SDS)-containing cell culture medium. An additional negative control contained hiPSC that were inoculated in cell culture medium without Y27632 dihydrochloride addition ($Y27632^{-}$). Error bars indicate mean ± SD, $n = 3$, $p < 0.05$.
